# *Aspergillus* diversity in the environments of nosocomial infection cases at a university hospital

**DOI:** 10.25122/jml-2018-0057

**Published:** 2019

**Authors:** Kambiz Diba, Farzaneh Jangi, Khadijeh Makhdoomi, Naser Moshiri, Fatemeh Mansouri

**Affiliations:** 1. Department of Medical Mycology and Parasitology, Faculty of Medicine, Urmia University of Medical Sciences, Urmia, Iran; 2. Cellular and Molecular Research Center, Urmia University of Medical Sciences, Urmia, Iran; 3. Imam Khomeini Hospital, Urmia University of Medical Sciences, Urmia, Iran; 4. Department of Genetics and Immunology, Faculty of Medicine, Urmia University of Medical Sciences, Urmia, Iran

**Keywords:** *Aspergillus*, identification, molecular, hospital

## Abstract

*Aspergillus* species (sp.) that causes opportunistic infections have been increasingly found in human mainly immunosuppressive patients around the world every year. The main objective was to use a rapid and cheap molecular method for monitoring *Aspergillus* infections and epidemiological approaches. In order to identity *Aspergilli* species (spp.), a number of molecular methods including restriction fragment length polymorphism (RFLP) have been employed in accordance with ribosomal RNA amplification. The focus of this study — a group of hospitalized patients with clinical and subclinical signs of infection. All of the collected clinical specimens were transported to the medical mycology lab and examined for *Aspergillus* identification. The environmental specimens were collected from air and surfaces inspected for the *Aspergillus* within the hospital sources. At first, growth characteristics and microscopic features on mycological media for the identification of *Aspergillus* sp. were performed. For the confirmation of *Aspergillus* isolates which similarly found in clinical and environmental sources, molecular method polymerase chain reaction/restriction fragment length polymorphism was carried out. From the mentioned specimens, 102 fungal isolates included *Candida* spp., *Aspergillus* spp. and other fungi. *Aspergillus flavus* (47%), *Aspergillus fumigatus* (29.4%) and *Aspergillus niger* (23.5%) all were found as the most common clinical isolates. In addition, *Aspergillus* isolates from environmental were *Aspergillus niger* (43.7%), *Aspergillus flavus* (41.7%), *Aspergillus fumigatus* (14.6%). Therefore, polymerase chain reaction-restriction fragment length polymorphism with a single restriction enzyme can be very useful in the identification of *Aspergillus* spp., because of its facility in use, speed, robust, and high sensitivity of diagnosis.

## Introduction

*Aspergillus* sp. falling under opportunistic fungi are often distributed and observed in soil, water, and decomposing vegetation. These species can also be isolated from unfiltered air, ventilation devices, dropped ceiling dirt, and polluted dust released from hospital renovation and construction operations. *Aspergillus* sp. may also colonize in catheters and implants [1]. As research findings suggested that these fungi underlay hospital-acquired infections (HAI) [2–5].

*Aspergillus* sp. are very profuse and most often found in soil, water, air, seed and, food. Some of these species can contribute to the development of a number of diseases including allergic bronco pulmonary disease, invasive infection, nasal sinusitis, mycotic keratitis, and otomycosis [11]. Aspergillus infection are developed through a path formed due to the inhalation of the fungal spores. And also patients with immunodeficient and immunosuppressed systems, pneumonia can be induced by local lung tissue invasion. In addition, the fungus may also spread into the bloodstream and affect various inner organs. Patients suffering from acute, prolonged granulocytopenia, especially those who undergo a bone-marrow transplant are most likely to be at the risk of developing filamentous fungi diseases, especially *Aspergillus* sp. Besides, patients diagnosed with aspergillosis are most likely to be infected by *A. fumigatus* and *A. flavus* which are regarded as the most commonly isolated species [3].

At least 30 species including *A. flavus, A. terreus, A. niger, A. nidulans, A.ustus* and *A.versicolor* underlie human diseases, while *A. fumigatus* is known as the most common cause of invasive aspergillosis. Therefore, choosing a reliable technique for early diagnosis of *Aspergillus* sp. leading to HAIs is essential and can contribute to monitor diseases — the effective use of epidemiological approaches. The early diagnosis of *Aspergillus* infections is a challenging task. During the past decades, with the increase in the number of antifungal agents, more drug resistant *Aspergillus* sp. have been developed [6–8]. The use of conventional microbiological, serologic, and imaging techniques do not lead to early diagnosis and effective treatment of Aspergillus infections. Clinical symptoms and signs and CT scan images can be employed to detect invasive diseases. Tissue invasion can be diagnosed by examining biopsy materials which enhances the chance of detecting invasive disease where the blood cultures are normally negative [9]. Besides, a set of clinical and pathological tests normally used for identification are time-consuming or less effective for routine surveys.

The chance of detecting HAI-inducing *Aspergillus* sp. can also be increased by employing a set of immunological and molecular tests [3, 9]. Molecular techniques which include the restriction fragment length polymorphism (RFLP) developed based on the amplification of ribosomal RNA have been used to recognize *Aspergilli* spp. and make effective treatment decisions [10]. PCR-RFLP method was employed in this study with a single restriction enzyme to identify *Aspergillu*s sp. isolated from HAI patients and the hospital interior environments. The advantages of this technique are its ease of use, cost-effectiveness, high speed, and its diversity power [6, 11, 12]. PCR-RFLP which is based on single restriction enzyme *Mwo*I has been proposed previously due to its reliability and accuracy than conventional techniques [13]. The data in this literature is insufficient for different countries with various circumstances. Therefore, this study addresses the identification of *Aspergillus* sp. in patients referring to a university hospital in Iran.

## Materials and Methods

### Clinical and environmental specimens

Most of the clinical specimens collected in this study are from patients suffering from acute infection symptoms hospitalized at a large university hospital. The invasive, acute disease was diagnosed using the clinical symptoms and CT scan images. Colonization of the patients was done by isolating the organism in the culture of specimens such as bronchiolar lavage, sinus discharge, sputum, synovial fluid, and urine. Our target group in this study included all patients with clinical symptoms of fungal infections 48–72 h after hospitalizing in the ward. All specimens were taken to the Medical Mycology Center, UMS University. Standard morphologic techniques were used to diagnose and identify *Aspergillus* elements as well as the growth characteristics and microscopic features of sabouraud glucose agar 4%. Besides, PCR-RFLP is a molecular method which is employed to identify isolated *Aspergillus* [13]. Additional specimens were collected for each positive case from places such as hospital interior spaces and equipment including air, surfaces of the walls and coverings, beds, sheets, trolleys, air conditioning, medical equipment and devices, finger touch samples of cases, staffs, and visitors. Air samples were taken by using sabouraud glucose agar 4% (SGA) plates left uncapped and exposed to air flow. Furthermore, the other samples were collected with sterile swabs and then were inoculated on a transport medium such as SGA 4% [12]. All samples including transport cultures were transferred to the Medical Mycology Center, Urmia Medical Sciences University (UMSU).

### DNA extraction

*Aspergillus* mycelia mass was prepared by filtering fungal liquid cultures for 12–24 h. Genomic DNA was taken from isolates by using glass beads in a lysis buffer (1 mM EDTA, 1% SDS, 100 mM NaCl, 10 mM Tris-HCl, 2% Triton X-100, pH=8.0). Then it was processed by the conventional phenol-chloroform method and was also checked by using agarose gel electrophoresis [14].

### PCR amplification

**Figure 1:**
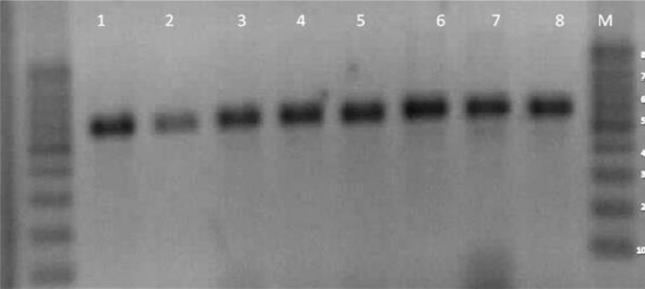
Agarose gel electrophoresis of amplified ITS fragments of *Aspergillus* sp. Lanes 1–8: *Aspergillus* isolates, lane M: 100 bp DNA ladder. As it shown, approximately all species make the same size PCR bands on agarose gel

Universal primers were used in this study to amplify *Aspergillus* ITS regions forward Primer: 5′- TCC GTA GGT GAA CCT GCG G - 3′ and Reverse Primer: 5′- TCC TCC GCT TAT TGAT TAT GC-3′. In addition, PCR assay was performed using 5 µl of the DNA template in a PCR buffer with the reaction volume of 50 µl (20 mM Tris- HCl, pH=8.0), 50 mM KCl, 0.1 mM each of forward and reverse primers for the ITS regions of ribosomal DNA, and 1.5 U of *Taq* DNA polymerase. All reactions occurred in a thermal cycler (XL model, Bioer, China). PCR program began by denaturing DNA at 95 °C for 5 min followed by 30 cycles. Each cycle includes three steps: denaturation, annealing, and extension steps at 95, 55, and 62 °C lasting 30 s, 1 min, and 5 min, respectively and a final extension at 72 °C for 5 min after the last cycle. Besides, a negative control, i.e., double deionized water (DDW, Merck, Germany), and positive control, DNA template extracted from standard *Candida* strain: *C. albicans* (ATCC 10261) (Boiling Phenol–Chloroform method), were employed. Electrophoresis was used to length separate DNA fragments via 1.5% agarose gels in Tris-Borate EDTA buffer and 0.50 mg ethidium bromide per ml. The results were documented using a tarns illuminator gel doc System (Figure 1).

### Digestion of PCR products by RFLP method

**Figure 2:**
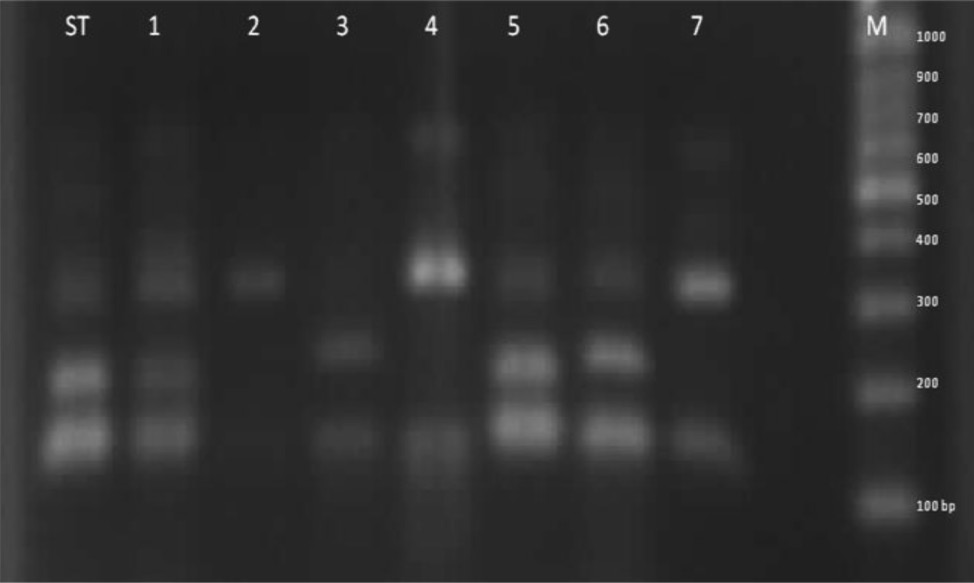
Agarose gel electrophoresis of ITS PCR products from standard *Aspergillus* sp. after digestion with the restriction enzyme *Mwo*I. Lanes ST: standard *Aspergillus* sp. as positive control, lanes 1, 3, 5, 6: *A. flavus*, lanes 2, 4, 7: *A. fumigatus*, Lane M: 100 bp DNA ladder

The restriction fragment length polymorphism technique was employed to produce differential patterns in order to identify *Aspergillus* spp.[14]. When amplified ITS fragments are digested with the proposed restriction enzyme, *Mwo*I at 37 °C enables differentiating some medically relevant *Aspergillus* sp. The same process was repeated for all clinical and environmental *Aspergillus* isolates. For the restricted digestion, 13 µl of each PCR product was directly digested by 5 U (0.5 µl) of the restriction enzyme, 1.5 µl of the 5X buffer, and was incubated at 37 °C for 180 min [15]. The digested PCR products were then treated with electrophoresis in agarose gel (2%) and visualized with gel doc system. In the last stage, *Aspergillus* sp. were identified in molecular terms by contrasting the electrophoresis bands with differential patterns (Figure 2).

## Results

The analysis of experimental findings for 198 clinical samples suggested that 93 (47%) of the cases were positive for the presence of fungal or bacterial infections. Besides, 54 (58%) positive cases had a fungal infection. This indicates that all of the isolated fungi can lead to nosocomial infections. The isolated fungi contained 36 *Candida* spp. (66.6%) and 17 *Aspergillus* spp. (31.4%). Besides, *A. flavus* (47%), *A. fumigatus* (29.4%) and *A. niger* (23.5%) were found to represent the most frequent clinically isolated *Aspergillus* sp., in descending order, as shown in Table 1. The black filamentous mold ‘*Alternaria alternata*’ was the only non-*Candida*, non-*Aspergillus* isolated fungus derived from a synovial sample of a patient suffering from septic arthritis. As it is shown in Figure 2, all identified samples were confirmed using PCR-RFLP technique.

**Table 1:** Identification of *Aspergillus* sp. isolated from clinical and environmental sources by RFLP method as well as their frequencies

	Clinical specimens		Environmental specimens		Total	
	No.	%	No.	%	No.	%
*A. flavus*	8	47	20	41.7	28	43.1
*A. niger*	4	23.5	21	43.7	25	38.5
*A. fumigatus*	5	29.5	7	14.6	12	18.4
Total	17	100	48	100	65	100

**Figure 3:**
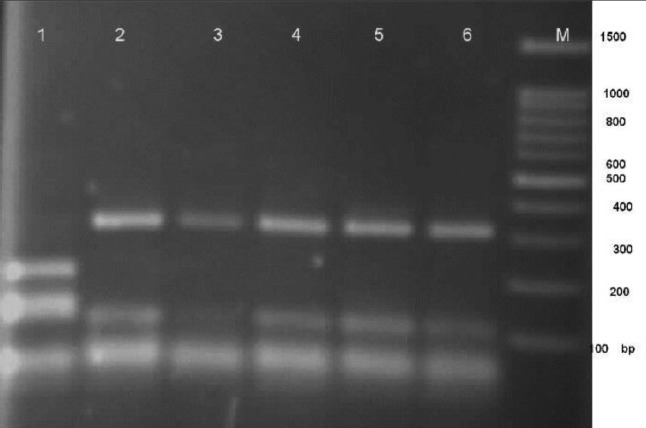
Agarose gel electrophoresis of ITS PCR products from clinical *Aspergillus* sp. after digestion with the restriction enzyme *Mwo*I. Lane 1: Positive Standard *Aspergillus nidulans*, lanes 2–6 *Aspergillus* group *flavi* and lane M: 100 bp DNA ladder

A total number of 256 specimens were collected from finger touches and body surface samples of the patients, hospital employees, and visitors, hospital devices and equipment including beds, floors, walls, trolleys, sinks, medical devices and air samples (Table 2). The samples collectecd containing 102 fungal isolates including *Candida* spp., *Aspergillus* spp., and other fungi as saprophytic molds: *Alternaria* sp., *Saccharomyces* sp., Mucorals, *Penicillium* sp., *Cladosporidium* sp., and Pheohyphomycetes.

**Table 2:** Isolation and frequency of opportunistic fungi in relation to hospital indoor contamination

Environmental specimens	Case	Staff	Visitor	Carpet	Walls	Bed and blanket	Sink	Trolleys	Medical devices	Air	Air conditioner	Out door
Contaminants												
*Candida*	1	5	1	2	4	4	3	1	0	0	0	2
*Aspergillus*	3	1	0	9	7	7	2	5	1	5	3	8
Others	0	0	0	3	5	4	1	4	6	4	1	0
Total	4	6	1	14	16	15	6	10	7	9	4	10

Among the total cases which were under study, 35 (31.5%) *Candida* spp., 48 (43.2%) *Aspergillus* spp. and 28 (20.3%) other fungi were isolated. In addition, the environmental *Aspergillus* isolates included *A. niger* (43.7%), *A. flavus* (41.7%), and *A. fumigatus* (14.6%) (Table 1).

## Discussion

There are many evidences to support the role of opportunistic fungi that act as main agents underlying in HAIs. For instance, in the time period from 1980 to 1990, *Aspergillus* spp. was identified as the main factor leading to life-threatening infections in immunocompromised patients [16]. *Aspergillus* elements are tipically isolated from soil, water, and compost. The spores have also been found in unfiltered air, air conditioning systems, and dust released through hospital renovation and construction activities.

A couple of molecular techniques were employed recently to discover opportunistic fungi such as *Candida* and *Aspergillus* sp. [17, 18]. As a case in point, the PCR-RFLP technique was employed by Moody and Tyler [10] to analyze interspecies variations of *Aspergillus* group *flavi* such as *A. flavus*, *A. parasiticus*, and *A. nomius*. Dendis et al. [19] also used the same technique to detect some pathogenic fungi in febrile neutropenic patients. RFLP was also used with a single restriction enzyme by Mirhendi and team [20] to identify and differentiate pathogenic fungi including *Candida* spp. The PCR-RFLP method with a single restriction enzyme, *Mwo*I*,* was also employed to discover *Aspergillus* spp. The whole molecular identification process including the isolation of the pathogen fungi from a short incubated culture, using DNA extraction, PCR, and RFLP profiles for 6–10 samples takes about several hours (12–18 h). However, the process lasts at least for 1–5 days with traditional methods. Therefore, this technique is faster than classic or morphologic identification approaches.

PCR-RFLP employing *Mwo*I was used in this study as a rapid and reliable technique to identify *Aspergillus* spp. released from clinical specimens. Accordingly, it was found that over 50% of all samples in the environmental isolates were positive in terms of the presence of at least one opportunistic fungus. The floor coverings, walls, and beds were the most frequent samples that were contaminated by the above fungi. This finding can be attributed to the fact that the patient cases had contacts with the above surfaces frequently and for a long time.

The process used to identify the isolated hospital fungi is of high importance as it contributes to select the most effective antifungal therapy. Furthermore, the use of unreliable techniques leading to misdiagnosis and overtreatment may speed up the spread of hospital infections.

Collecting hospital fungi isolates from cases and employees’ finger touch samples is important (Table 2). *Aspergillus* spp. representing the second most important group of fungi underlying nosocomial infections was sampled from the air and indoor/outdoor places at the hospital. The most contaminated hospital indoor samples were collected from carpets, walls, and also air specimens in the hospital rooms [2]. *Aspergillus* spp. is inhaled mainly from the spores existing in aerosols suspended in the air. However, the present study did not address the isolation of *Aspergillus* spore from air samples. Respirable fungal spores can be released from building or construction projects and spread into the environment [1]. Because of their small size, *Aspergillus* spores can suspend in the air for a long time. Therefore, the use of antiseptic agents (fungicides) on a daily basis as a routine laboratory procedure is recommended.

Our results of *Aspergillus* isolation are in agreement with the above studies implying that *A. fumigatus* and *A. flavus* are the most commonly isolated *Aspergillus* spp. in the patients with diagnosed aspergillosis. Molecular assays that allow genetic characterization of *Aspergillus* phenotype diversity are likely to become incorporated into major mycologist guidelines that are widely applicable in clinical practice. In addition, early identification is important for discovering new antifungal drugs to control hospital and environmental infections [21].

## Acknowledgements

The authors would like to thank the deputy student services for their kind help to collect the samples. This study as a scientific project (contract number: 1395-01-32-2781) was financed by the Research and Technology Deputy of Urmia University of Medical Sciences.

## Conflict of Interest

The authors confirm that there are no conflicts of interest.
